# Retraction: Racial Disparity Amongst Stroke Patients During the Coronavirus Disease 2019 Pandemic

**DOI:** 10.7759/cureus.r21

**Published:** 2021-01-13

**Authors:** Hammad Ghanchi, Tye Patchana, James Wiginton, Jonathan D Browne, Ai Ohno, Ronit Farahmandian, Jason Duong, Vladimir Cortez, Dan E Miulli

**Affiliations:** 1 Neurosurgery, Riverside University Health System Medical Center, Moreno Valley, USA; 2 School of Medicine, California University of Science and Medicine, Colton, USA; 3 Neurosurgery, California University of Science and Medicine, San Bernardino, USA; 4 School of Medicine, California University of Science and Medicine, San Bernardino, USA; 5 Neurological Surgery, Arrowhead Regional Medical Center, Colton, USA; 6 Neurological Surgery, Riverside University Health System Medical Center, Rancho Cucamonga, USA; 7 Neurological Surgery, Riverside University Health System Medical Center, Moreno Valley, USA; 8 Neurosurgery, Arrowhead Regional Medical Center, Colton, USA

This article has been retracted as it includes data published without permission from the Get With the Guidelines database, which is property of American Heart Association (AHA). Although the authors' hospital had access to this database and they did receive IRB approval, the terms of use between the hospital and AHA do not allow publication of the data in a public sphere. The authors were hoping to receive retrospective approval from the AHA but have not been successful in getting this permission thus far.  As a result this article has been retracted at the request of the AHA and with full agreement by the authors.

